# Emotional foundations of the market: Sympathy and self-interest

**DOI:** 10.3389/fsoc.2022.1054291

**Published:** 2022-11-14

**Authors:** Emiliano Bevilacqua

**Affiliations:** Department of Human and Social Sciences, University of Salento, Lecce, Italy

**Keywords:** market, emotion, sympathy, self-interest, Smith, Weber

## Abstract

Sociology shows the role of emotions in economic life. Sympathy and self-interest are crucial individual dispositions to explain the social behavior that shapes market institutions. Adam Smith emphasized the importance that sympathy has in the achievement of stability in social interactions that foster market society. On the other hand, Max Weber argued that disciplined self-interest is essential for the accumulation of capital. Although their analyses differed in some aspects, both Smith and Weber considered emotions to be the key to understanding the moral values that drive economic behavior. This paper will compare Smith's and Weber's theories of the relationship between emotions and the market. Finally, this paper will interpret sympathy and self-interest as the emotional foundations of the market, highlighting the fundamental role that emotions might have in economic analyses.

## Introduction. Emotions and the market

The awareness that rationality cannot completely explain the complexity of social behavior has allowed social sciences to overcome a first positivist phase (Elster, 1979; Simon, 1983). Unlike neoclassical economics, which assumes the individual to be rational and able to act based on a cost-benefit analysis of actions, sociology focuses on emotions, seen as concurrent causes of one's actions. Emotions are not considered to be mainly individual dispositions, but they are analyzed as social variables, based on the idea that the culture of a specific historical period influences the inner reality of individuals and their various emotions, from sympathy to emulation (Barbalet, 2002; Turner and Stets, 2005).

Market rationality has been the subject of multiple studies that have resulted in conflicting and controversial findings. Although the different forms of rationality and their direct impact on the modern economy will not be examined in this paper, it will be argued that emotions shape both the market and its institutions. Therefore, this paper will focus on both the impact of emotions on the economy and the models of subjectivity that characterize the market, in order to highlight the social variables that influence economic life. Smith analysis (1904; 1978; 1984) and Weber's reflection (1922, 2005) shed light on emotions such as sympathy and self-interest, outlining important models of subjectivity in the history of capitalism.

Smith's theories represented a turning point in the history of thought, as he considered social interaction to be at the origin of specific moral principles that were congruent with the interests of the market by virtue of being based on sympathy. The second section of this paper will focus on Smith's theories and will discuss the process of emotional identification supporting economic life. The third section will deal with the emotional implications of Protestant ethics arising from the Calvinist concept of self-interest, highlighting how the believer's inclination to production derives from an asphyxial emotional disposition and extremely poor social interactions. Weber described a model of subjectivity that is strongly dependent on society and history. Finally, this paper will investigate some implications derived from the relationship between emotions and the market, pointing out how emotions play a key role in balancing contradictory economic trends, in terms of both production and consumption. Smith and Weber interpreted emotions as a social phenomenon, thus emphasizing the link between the history of the market and a change in subjectivity.

## The impact of sympathy on the balanced dynamism of the market

The individual's sympathetic tendency represents the most significant dimension of Smith's sociology, acting as an explanatory factor of the origin and physiology of the market. The most innovative aspect of Smith's reflection is his idea of human nature, which differs from negative anthropology characterizing English Contractualism (Dwyer, 1998, p. 3; Hont, 2005, p. 37 ff.; Berry, 2013, ch. VII). In the late 18th century, the Scottish reflection on the self abandoned a metaphysical and philosophical approach to embrace the idea that the foundation of human nature lies in social interactions (Skinner, 2004). As character is always “situationally dependent,” the “understanding of self was the empirical base for our knowledge of sociability” (Ahnert and Manning, 2011, p. 8).

According to Smith, the individual's emotional nature leads them to a sympathetic disposition toward other human beings, affecting both their inner reality and social interactions. Human beings identify with their counterparts not only in the processes of socialization characterizing everyday life, but also when interacting with institutions. Recent studies have interpreted Smith's concept of sympathetic disposition in light of the neuroscientific theories of empathy and its social implications (Boyd, 2013; Kelly, 2013). Having analyzed sympathy in light of the theory of mind, Sule Özler and Paul Gabrinetti have stated that “the process of sympathy in Smith was a forerunner of empathy in the modern psychoanalytic literature” (Özler and Gabrinetti, 2018, p. 17). However, this paper will focus on the sociological dimension of Smithian emotions and its capability to explain important socio-economic phenomena. The Scottish Enlightenment, of which Smith was a member, paid great attention to the sociological importance of emotions. “A major insight of the enlightened writers was the primary role which the human emotions played in individual motivation and social organization. While reason enabled observers to better understand the workings and significance of the human passions, it was the latter rather than the former which were the ‘springs' of group life and the primary platform upon which progress proceeded” (Dwyer, 1998, p. 1).

Smith's focus on sympathy—regardless of its degree of convergence with empathy—represents the best example of an analysis that acknowledges the important role emotions play in influencing individual behavior and moral values. Individuals feel gratified when being rewarded and appreciated, and at the same time they emotionally understand other people's experiences, especially when witnessing them. As Smith pointed out, “how selfish soever man may be supposed, there are evidently some principles in his nature, which interest him in the fortune of others, and render their happiness necessary to him, though he derives nothing from it except the pleasure of seeing it […] [N]othing pleases us more than to observe in other men a fellow-feeling with all the emotions of our own breast; nor are we ever so much shocked as by the appearance of the contrary” (Smith, 1984, p. 81, 89). Smith suggested a double interpretation of sympathetic subjectivity, which may express itself in both an individual request for emotional recognition and a social propensity to cooperation. However, despite describing human nature as being emotionally permeable, the Scottish sociologist did not link it with a necessarily benevolent subjectivity (Zanini, 2014, p. 97 ff.). Emotions emphasize the social dimension of human beings and, consequently, significantly contribute to emotionally characterizing Smith's sociology. After all, the Scottish sociologist had no intention of replacing Hobbes's notion of human nature with a contrasting, idyllic perception of human beings. Smith identified the individual's emotional tendency toward other human beings, which became the basis for his interpretation of social life. The individual's emotional propensity is described by virtue of an immanent interpretation of social life—which is far from philosophical metaphysics—and results in the individual's social nature being reclaimed (Packhman, 2012). Emotions are an aspect of social life that may have different characteristics, based on the historical and social factors determining human interactions, which become rooted in institutional life. From such a perspective, sympathy seems to play a key role, since “[s]ympathy was a social practice through which individuals who share physical space participate together in an ordinary exchange of approbation and shame, and through repetitive interactions over time learn to become ‘social'—learn to adjust their passions to a ‘pitch' commensurate with living in a society with others” (Forman-Barzilai, 2009, p. 12–13). The social nature of individuals is reclaimed, although within an immanent analysis that examines its relational development in everyday life.

Therefore, sympathy has an unavoidable moral impact. Both the mutual recognition pursued during an interaction and the emotional mediation necessary to express one's identity by achieving socially legitimate objectives result in values being shared regardless of political or legal norms. Smith considered values and norms to be the product of a slow process of institutional development, a consequence of the network of everyday interpersonal relationships affected by the emotional disposition of human beings. Morality develops from civil life in order to achieve a social and political institutionalization (Fitzgibbons, 1995).

In this sense, it is not surprising that Smith lets reason and emotions coexist. Rationality is a distinctive feature of the human being, together with the subjective tendency to wish for recognition and perceive the other's expectations. According to Smith, the individual is both rational and able to act based on the emotions arising from interactions. Emma Rothschild and Amartya Sen have pointed out such an aspect when stating that “rationality is in general, for Smith, a sociable and discursive condition. Reasoning and conversation are companions” (Rothschild and Sen, 2006, p. 362). Smith's analysis of rationality shows his contribution to a sociology of emotions that can help to explain society and economic institutions such as the market. Although institutions develop, change and multiply based on human rationality, their social usefulness derives from providing a social space for the expectations of recognition and interaction that enliven the emotional nature of human beings. The coexistence of rationality and emotions envisages a morality whose strength is directly proportional to the depth of the process determining its development. Smith identified moral values that can be said to be reasonable due to their benefiting the community, being emotionally gratifying and institutionally legitimized by virtue of their widespread relational experimentation.

The social organization that emerges from this analysis of human nature is characterized by individuals who tend to identify with one another, producing collective behavior that allows for a sociological investigation of social institutions. From this perspective, the market is no exception. Smith interpreted economic life in light of the individual's tendency to identify with other human beings through processes of socialization and everyday practices of interaction. Human imagination is not defined as a subjective factor, but rather as a social disposition that enables an individual to emotionally understand and morally introject the other's experiences, thus contributing to a shared culture (Kelly, 2013; Zanini, 2014, p. 47 ff.). Imagination proves to be essential to explain the individual's tendency to emulate, which becomes crucial in the everyday social interactions that shape market society. The desire to emulate one's prosperity and prestige has a significant role in economic life. Furthermore, the Smithian notion of the impartial spectator can be sociologically interpreted as an inner consequence of such a social process (Montes, 2003, p. 86 ff.). According to Smith, this category refers to the individual's sense of moral responsibility toward society, which is expressed by the unbiased inner judge within every citizen. The impartial spectator reveals the result of the mediation between individual expectations and institutional obligations (Keppler, 2010, p. 65 ff.). Smith intended to emphasize the possibility for individuals to share common values that allow them to pursue individual interests without renouncing the common good. He pointed out how the habit of self-reflection results in a prudent attitude enabling to strike a balance between individual passions and social obligations (Singer, 2004; Zanini, 2014, p. 136 ff.).

Smith's ethics is based on the possibility of embracing and reconciling both the pursuit of individual freedom and the protection and development of the social order. The achievement of such an objective is the result of the individual's emotional propensity and ability to derive common moral principles from that. Observing the market from this perspective shows how the individual's identification with other human beings benefits from imagination and the concept of the impartial spectator, resulting in a model of subjectivity that encourages individuals to adopt a balanced dynamism, based on both the desire to distinguish oneself and the respect for a good development of society.

Sympathetic identification is therefore the social precondition of the market, which, in turn, gives an economic value to the individual's qualities and social interactions. Although there is no need to emphasize Smith's role as a forerunner of an institutional approach to economic sociology, it seems essential to point out that he understood the need for an institutionalization of the stability of economic life (Trigilia, 2002, p. 19 ff.). Anomic market trends were already evident in the late 18th century, especially in relation to the alienating impact of the division of labor. Smith argued that the strength of the new, developing economic system lay in its promise of individual and universal success. However, he also understood the risks posed by a process of unconstrained accumulation, incapable of mediating between individual claims and the need for social integration.

The emotional origins of the market and the role played by emotions in balancing the capitalist tendency to accumulate are the most significant aspects of Smith's sociological analysis of economic life. Reference to the problem of the origins is made in both *Glasgow Lectures* and *The Wealth of Nations*. In both works, the individual's emotional and relational attitudes are regarded as determining factors for trade and its institutionalization. Rather than embracing Marx's focus on the privatization of common goods (Marx, 1867-1885-1894), or Durkheim's idea of the key role played by the division of labor (Durkheim, 1967a,b), Smith identified the tendency to barter as the origin of the market, showing how such tendency represents a historical variation of the more general propensity to persuade and to establish relationships that guides social interactions. According to Smith, “[i]f we should enquire into the principle in the human mind on which this disposition of trucking is founded, it is clearly the natural inclination everyone has to persuade” (Smith, 1978, p. 352). Therefore, Smith associates a disposition to trade with socio-emotional interactions and the individual's need for recognition. Social interactions, and hence the relational attitude originating from the individual's emotional disposition, play a causally significant role in the development of modern economy. Although Smith also described the historical conditions allowing for an effective institutionalization of such an attitude, what really enables to evaluate the emotional foundations of the market is the Smithian focus on the individual's tendency to establish relationships with other human beings, in order to achieve that intersubjectivity that emerges at a social level, which led to the first institutionalization of the market in the 1750s.

Smith's reflection intertwines the discussion on the origins of the market with the analysis of the role emotions have in the routine of commercial society. Smith pointed out how sympathy-oriented individuals establish mutual relationships and, with some specific institutional conditions being present, encourage a collective behavior that balances individual interests and the moral values necessary to stabilize the market. It seems important to highlight Smith's ability to show the most distinctive feature of the balanced dynamism that characterizes the market in some specific circumstances. Smith attached an emotional connotation to economic freedom, being fully aware that the market is founded on the daily reality of individuals who pursue their own satisfaction by searching for emotional recognition in social interactions (Honneth, 2018). Concurrently, the economic system needs a moral order, and hence individuals who are able to limit their interests through moral principles that, by fostering prudence and virtue, consider work the main tool to achieve personal success and social wellbeing. The continuity of work proves to be essential in the development of a process of production and accumulation of capital. This is true for both workers, who should be able to search for their satisfaction in the social form of wage labor, and capitalists, who should control the satisfaction they find in accumulating wealth in the social form of capital investment. Smith argued that economic wellbeing depends on particular character traits that allow individuals to reject strong, anti-social individualism and embrace moral principles that legitimize a social regulation of the market through rules that reduce the strength of individualism (Hirshman, 1977).

Therefore, managing emotions is instrumental in institutionalizing the market. The tendency to mutual persuasion, the search for recognition, and the sense of fulfillment gained from the exchange of goods and services enable individuals to express their personality and pursue their interests, while also allowing them to participate in a social order whose morality is accepted by virtue of being remarkably widespread and socially legitimate. The market thus expresses individual volition through a system of rules that limit its excesses. Such dynamics ensure economic reproduction, as they discourage deviant behavior and prevent moral disorder (Singer, 2004).

The spirit of capitalism, just to make reference to Max Weber's expression, lies in a sympathetic subjectivity that acts rationally through the individual's identification with other human beings, so as to lead modern society toward a dynamic, although balanced, economic system. According to Smith, emotions prove to be essential as they restore the strength of a subjectivity that rejects the moral imperatives of a declining traditional order. Although rationality is not considered to be as important as it was in Cartesian anthropology, it acquires more realistic features, corresponding to an immanent analysis of human nature. Self-interested individualism feeds on a rational cost-benefit calculation in the pursuit of individual objectives. However, as Smith demonstrated, it has its roots in the individual's emotional disposition and is remarkably widespread, by virtue of the prudent morality emerging from social relationships established by individuals who are unburdened by traditions.

Therefore, emotions convey a morality that puts individuals at the core of a new social order. Such an aspect has been emphasized by Emma Rothschild, who has argued that “[t]he discursive political society, or the philosophical politics of good-tempered discussion, requires a society of good-tempered, prudent moral sentiments; this is the condition for civilized conflict […] [t]he system of commerce requires, as has just been seen, a system of moral sentiments, or a good-tempered, prudent society; this is the condition of civilized competition” (Rothschild, 2001, p. 245). The market expresses such a reality, as it achieves an increasing collective wellbeing while leaving considerable room for maneuver to the individual. Emotions influence the market in terms of both its origin and physiology, especially through sympathy. First, sympathy encourages individuals to interact, fostering barter, and at a later time it unites them through common values that become rooted in society, legitimizing new economic practices. Such a virtuous circle depends on the emotional nature of human beings and their sympathetic interactions. Smith's pioneering anthropological theories focused on the role that individual emotions and their sharing play in modern economic life, while highlighting the need for an institutional control of capitalism.

## The foundations of economic rationalism: Regulation of emotions and utilitarian self-interest

The late-18th-century developing market allowed Smith to grasp the ambivalence of a new economic system that, despite being impacted by an alienating division of labor, fostered the economic growth of society and resulted in individual freedoms. Although both social progress and the process of individualization are characterized by controversial and contradictory features, they have played a key role in the socio-economic history of modernity, being fascinating still nowadays.

On the other hand, in the early 20th century, Max Weber observed a more mature modern economy. This seems to explain why Weber's analysis is less optimistic than Smith's, being more focused on highlighting the alienation and conformity caused by monopolistic capitalism (Kaesler, 1998). However, both Weber and Smith carried out a historical analysis of the market and suggested a correlation between economic development and individual emotions.

While in *Economy and Society* (2019) Weber introduced a type of social action that also includes the affectual element (Fitzi, 2022), *The Protestant Ethic* shows the effectiveness of a cultural explanation of economic life. As Charles Turner has pointed out, “Weber understood culture less in terms of the need to defend a sense of inwardness and depth against the encroachments of modern bureaucracy, than as a means of conceptualizing the relationship between the unity and differentiation of the personality, the ordeal of determinate human conduct as such” (Turner, 1992, p. 3). Weber's research into the ethics of monotheistic religions drew attention to the cultural origins of the most significant social phenomena of modernity—rationalization and utilitarian self-interest (Eisenstadt, 1968). According to Weber, the loneliness resulting from a Calvinist fear of death, caused by God's inscrutable will, pushes individuals—in European areas characterized by specific socio-economic conditions—to search for the signs of their predestination in economic success, an assumed proof of uncertain salvation. This shows how a religious-based cultural change contributed largely to the rationalization of the economy and the accumulation of capital. Although the complex issues of Calvinist theology influenced the analysis of the relationship between emotions and the market only relatively, a particular aspect of the Reformation might clarify the model of Calvinist subjectivity and its contribution to economic life. The Calvinist belief that human actions cannot influence God's will paved the way to the disenchantment of the world and, destroying the social foundations of magic, laid the groundwork for the legitimation of rational asceticism (Schluchter, 1991). The disenchantment of the world is fed both by the idea that the worldly order inevitably shows God's will and by the belief that every individual is nothing but a cog in the divine rule over life on earth. The conformity resulting from such an outlook shapes personalities that are naturally in line with the social order and, being influenced by the development of the market, envisages a society that supports capitalism and legitimizes a utilitarian worldview. The Protestant *Beruf* therefore “[…] expresses the value placed upon rational activity carried on according to the rational capitalistic principle, as the fulfillment of a God-given task” (Weber, 1950, p. 267).

Weber focused on such aspects to interpret the origins and physiology of the market. Initially characterized by a moral attitude to work and accumulation aimed at attaining salvation, Calvinist ethics gradually evolved into an endless, senseless production routine. Such an evolution has its roots in the heteronomy of purposes that characterizes life conduct. As Weber argued in *The Protestant Ethic*, the heteronomy of purposes transforms a moral search for salvation into a progressive legitimation of the instrumental rationality of the market, seen as a chosen space for the most deserving. Calvinism abandoned its initial moral strength—resulting from the believer's loneliness before an unfathomable afterlife—to embrace utilitarian self-interest, the main path to the achievement of economic wellbeing, considered to be an unmistakable sign of predestination. The pursuit of economic objectives based on a rational cost-benefit calculation became the main logic in social behavior. This is a good example of the heteronomy of purposes often connected with collective behavior (Merton, 1936). The final outcome of such a process is a society in which rationality loses its nature, trapping individuals in the iron cage of deeply rooted but unreflective behavior, rather than helping them to freely decide about their life (Hennis, 1987; Brubaker, 2010). These are the unintentional consequences of a cultural process that Weber considered to be essential to explain economic and social life.

Weber insisted on the emotionless nature of Calvinist subjectivity, attributable to the spread of an instrumental rationality that fails to ponder on the meaning and purposes of action, but translates into a natural convergence of the individual's morality and the market system. Therefore, Weber wondered about the individual behavior encouraged by the Reformation. His analysis of social relationships within Protestant sects shows the emotional orientation of Calvinist ethics (Poggi, 1983). Weber was impressed by the fact that the bonds forged through a common faith bring with them a strict control on the believer's private life, while hindering the establishment of emotionally significant relationships. Such an aspect greatly impacts the shaping of personality, as “the social organization of the sects had provided the means by which the ethical teachings of Puritan religiosity had become inculcated in a methodical style of life” (Bendix, 1960, p. 89). Weber observed that such an emotional self-control seems to be inconsistent with the emotional sharing characterizing religious groups. The emotional illiteracy of Calvinist believers is correlated with the spread of instrumental rationality and the consequent individual acceptance of the socio-economic routine of the market.

The contradiction shown by the analysis of Protestant sects, the oxymoron of emotionally neutral communities of believers, emphasizes the model of restrained subjectivity typical of the early 20th century, besides substantiating the Weberian homology between life conduct and economic change. According to Weber, individuals whose personality is characterized by self-discipline and self-control tend to establish impersonal relationships. Such an aspect marked social interactions in the areas affected by the Reformation, outlining the personality traits of the bourgeoisie. The historical circumstances led to the theological foundations of Calvinist ethics being translated into the pragmatic language of economic development. The religious legitimation of the social order and the skepticism about the possibility of changing essential social aspects caused a stigma to be attached to emotions, which began to be regarded with suspicion, as they tempted individuals into deifying a human being, distracting them from the accomplishment of the worldly tasks set by God. The emotional investment in relationships was strongly condemned, as it celebrated the human being, leaving little room for God. Insisting that such a process of personality development might impact social interactions, Weber stated that “[b]rotherly love, since it may only be practiced for the glory of God and not in the service of the flesh, is expressed in the first place in the fulfillment of the daily tasks given by the lex naturae; and in the Process this fulfillment assumes a peculiarly objective and impersonal character, that of service in the interest of the rational organization of our social environment” (Weber, 2005, p. 64). By disapproving of the arts, friendship, and sexuality, Calvinist theology confirms such a perspective. The imperative of silence, a fundamental aspect in Reformed theology, is yet another prohibition imposed by the restrained subjectivity and impersonal society Weber described.

Weber's interpretation of the Calvinist decision to abolish the Sacrament of Penance proves to be an example of the work he carried out to translate individual psychology into sociology, showing the origins of emotions and their role in the development of the market. The Rite of Penance allows believers to communicate with God and relieve their anxiety about sin, easing their concerns about their fate in the afterlife. However, Calvinism prevents individuals from sharing the sense of guilt for their sinful behavior, as priests are no longer allowed to mediate between human beings and God, with believers losing the possibility of receiving forgiveness through absolution. As a result, the surplus of energy produced by worrying is channeled into strict, systematic, self-disciplined behavior (Weber, 2005, p. 69 ff.). Such a psychological outlook ends up inhibiting social interactions, prioritizing self-control and impersonality over one's emotions. Therefore, not only are emotions an important aspect of subjectivity, but they also play a social role. Protestant ethics transforms emotional energy into a systematic search for economic success, which is the only sign of predestination. The utilitarianism that characterizes the market has its roots in the relationship that developed between culture, ethics and economy in Modern Europe.

It is now easier to understand how emotions have played an important role in the process that leads from the disenchantment of the world to instrumental rationalization and the market. Calvinist precepts affect emotions by increasing the individual's concern and anxiety, thus becoming a factor of emotional restraint and redirecting the individual's energy only to self-interest (Bericat Alastuey, 2001; Marcuse, 2009). Such a socio-cultural process is in line with the capitalist need for an immediate reinvestment of accumulated capital. This helps to both boost production, by offering cutting-edge goods and services, and protect profit margins, thus preventing any crisis. The cyclical nature of a capitalist economy can be regarded as both the endogenous pressure for accumulation and a social urge for productive activity (Horkheimer and Adorno, 1947; Marcuse, 2009, p. 151 ff.). As the “worldly Protestant asceticism […] acted powerfully against the spontaneous enjoyment of possessions; it restricted consumption, especially of luxuries” (Weber, 2005, p. 115), the capitalist pressure to indefinitely increase individual wealth and collective wellbeing fosters the development of a self-interested personality that is totally and indefinitely focused on work and accumulation (Müller, 2005-2006, p. 136 ff.). The omnilateral human nature becomes bound to economic instrumental utilitarianism, while other needs and objectives, including emotional ones, lose their significance. The individual's instrumentally rational action becomes the main point of reference (Weber, 2001). Discussing such a phenomenon helps to understand how the social processes related to the market might constrain emotional attitudes, their moral value and social legitimacy.

The duty to behave in a religious manner results in pleasure and leisure time being stigmatized, as they are considered to be an undeserved glorification of the individual. As in the Modern period money was a factor of social differentiation (Simmel, 1900), and hence an instrument of individual enjoyment and freedom, the inhibited Calvinist changed the relationship between money and society. People were led to believe that one's wealth should not be used to increase individual experiences and opportunities, but should be totally reinvested in production, the only way to confirm the signs of economic success, and hence the signs of predestination, for the greater glory of God. The objective of continuously generating money, after all, is in line with the idea of the market as the chosen field for the objectification of the world through a rational exchange of values (Scaff, 1989; Rehmann, 2013, p. 326 ff.). Individuals reject magic by focusing on the calculable nature of their investments and, concurrently, they distance themselves from the emotional aspects of money making, subordinating ruthless greed, the waste of goods, and leisure time to the rationality of production (Bataille, 1967, p. 155 ff.). Reinhard Bendix has effectively summarized the social context Weber described and its impact on subjectivity by stating that “in the case of Puritanism he noted certain explicit demands and values: the negative attitude toward art, sexuality, and friendship; the rejection of all magic and symbolism, of the confessional, and of burial ceremonies; the contemptuous attitude toward poverty and the poor; the distrust of human relations but the reliance on impersonal probity” (Bendix, 1960, p. 278–279). The assessment of the profitability of capital and all the accounting tools created in the Modern period subordinate work to accumulation and develop a new kind of productive rationality that shapes individual behavior. Therefore, Weber described a rational economic system that, paradoxically, has its roots in a particular emotional attitude that is focused on individual self-interest. Influenced by the subordination of one's emotions to the rational imperatives of interest, the social regulation of emotions is caused by Calvinist moral inhibitions being channeled into practical activities.

The Protestant propensity to defer gratification by delaying the enjoyment of wealth and pleasures provides an example of how one's wishes are restrained, being sacrificed on the altar of capital reinvestment and continuous economic success. The fact that frugality became part of the way of life of the ruling class not only marked a change in social history, but it also showed the role played by the regulation of emotions in modern economic life (Sombart, 1913). The deferral of gratification subordinates the emotional aspects of personality to instrumental rationalism, shaping an anthropological model that sacrifices the emotional and all the other dimensions of subjectivity to economic logic. Weber pointed out how it is the internalization of Calvinist ethics that makes it possible for individuals to have the constant self-control that is necessary to restrain the daily emotions that might otherwise compromise a regular economically rational behavior. The cultural change brought about by Calvinism resulted in a social process capable of transforming individuals, suppressing their emotional disposition and directing them toward the market. According to Weber, the outcome of such a change leads individuals to forget the religious origins of Protestant ethics, to unconsciously build a world that lacks any sense of action, being totally subordinated to relentless and unintentional economic activity: “In fact, the summum bonum of this ethic, the earning of more and more money, combined with the strict avoidance of all spontaneous enjoyment of life, is above all completely devoid of any eudæmonistic, not to say hedonistic, admixture. It is thought of so purely as an end in itself, that from the point of view of the happiness of, or utility to, the single individual, it appears entirely transcendental and absolutely irrational. Man is dominated by the making of money, by acquisition as the ultimate purpose of his life. Economic acquisition is no longer subordinated to man as the means for the satisfaction of his material needs (Weber, 2005, p. 18).

## Concluding remarks

Adam Smith and Max Weber emphasized the role played by emotions in the development of a social context and a subjectivity that foster market economy. Smith showed how sympathy facilitates both the expression of an independent, enterprising subjectivity and the establishment of orderly, cooperative social interactions capable of limiting individual interests. Weber focused on such an aspect in order to show how restraining emotions underlies self-interested patterns of social behavior that are constantly oriented toward the monotonous regularity characterizing the pursuit of economic profit.

The so-called “Adam Smith Problem,” which arose from a seeming inconsistency between *The Wealth of Nations* and *The Theory of Moral Sentiments*, revolved around the correlation between the enterprising, selfish propensity of the economic individual and the sympathetic, altruistic tendencies of mid-18th-century society (Montes, 2003). However, when such a debate was brought to a close, it became clear that Smith considered the individual's cravings and feelings to play a significant role in both the economic sphere and daily life, being restrained and controlled by socialization processes and social morality. On the other hand, the controversial theories that Weber developed in *The Protestant Ethic and the Spirit of Capitalism* have been repeatedly criticized. Showing how even pre-16th-century European society could already be regarded as capitalistic, Trevor-Roper has explored the reasons that led to a new, different concentration of the most dynamic economic actors in specific areas. Furthermore, Samuelsson has highlighted how religious beliefs, including Protestantism, have constantly discouraged the accumulation of capital. Other scholars have expressed doubts about sources (Novak, Sombart). However, Weber's analysis of the emotional restraint of economic subjectivity and the role played by emotions in the development of bourgeois subjectivity has not been the object of criticism.

Therefore, both scholars ascertained how the physiology of the market is facilitated and fostered by the changes that take place in the field of emotional socialization. Smith's analysis demonstrated how Modernity broke with traditional roles to pave the way to a socio-cultural dynamism in line with the active economic nature of the market. On the other hand, Weber's works showed how the possibility of improving one's status opened up by Modernity translates into a social tendency to redirect most of the individual's emotional energy into a stable, systematic disposition to work and invest. Such a reflection led Weber to point out how the rational behavior accompanying the market, caused by that state of concern and anxiety typical of the Calvinist doctrine, evolves into instrumental self-interest with strong emotional connotations. Weber's interest did not lie as much in the cognitive impact of religious precepts, but rather in their emotional consequences and their influence on social action and economy. Thus, the more relative importance of rationality strengthens the sociological criticism of the neoclassical paradigm, contributing to a social explanation of economy, in terms of both understanding the role played by emotions and denouncing the controversial impact of instrumental rationality on the individual.

As Mizuta (1975) has pointed out, both Smith and Weber discussed the relationship between religion and economy, with particular reference to the role played by religious precepts in suppressing individual wishes and channeling one's energy into entrepreneurial activity (see also Barbalet, 2008, p. 111 ff.). Although Smith emphasized the role of emotions in the development of a subjectivity that is open to the other, he also highlighted how the processes of socialization can promote a cautious attitude that pushes individuals to accept socially legitimate customs and traditions. On the other hand, Weber's inner-worldly asceticism shows how the pursuit of personal interest encouraged by the Calvinist legitimation of money resulted in a conformity of behavior that, in the early 1900s, seemed to reject all the most innovative and heterodox aspects of bourgeois subjectivity. Therefore, Weber's works focus on the conformist introversion of emotions in order to highlight how, with the development of capitalism, self-control directed toward work and accumulation, the Calvinist escape from the prospect of damnation, became a social automatism devoid of any moral foundations. By contrast, there was an increase in the propensity to defer gratification so as to sacrifice it on the altar of accumulation, which is the result of that regulation of emotions that Smith described in the late 18th century and Weber considered even more significant in the early 1900s.

When discussing some of the social phenomena investigated in Smith's works, David Reisman has stated that “all of this was to be perceived subjectively: like Max Weber, Smith was passionately concerned with the subjective meaning that human interaction had for the individuals involved” (Riesman, 1976, p. 11). Although Smith's and Weber's analyses of the emotional impact of social interaction differed, in the same way as the historical contexts they were experiencing differed—Smith's being more open to emotional social interactions and Weber's being more focused on the control of emotions—both Smith and Weber showed how emotions influence economic life and the individual, helping to understand collective behavior and moral values. *The Theory of Moral Sentiments* and *The Protestant Ethic* highlight the presence of social rules that regulate social interaction and emotional introjection, shaping their forms of expression. A number of such aspects are still present in contemporary society, as it has been demonstrated by the various transformations of subjectivity that have characterized globalization processes and reshaped the economy, from the commercialization of intimate life to the inclusion of leisure time and emotional communication in the economic sphere of production and consumption (Lasch, 1984; Beck and Beck-Gernsheim, 1990; Giddens, 2003). The sociology of emotions has thoroughly investigated these topics, whose analysis has contributed to directing the attention of social sciences toward emotions (Hochschild, 1979, 1983, 2003). Eva Illouz has pointed out how “Max Weber has most compellingly described the relationship between entrepreneurial capitalism and the personality structures that made it possible” (Illouz, 1997, p. 188). On the other hand, as Berezin (2009, p. 342) has stated, classical theorists, including Adam Smith, feared the unpredictable impact that emotions might have on behavior. The social processes of late capitalism, such as the success of the cultural industry, the increased importance of leisure time, the monetisation of the human body and of sexual identity, have emphasized how the relationship between emotions and the market theorized by classical sociology has influenced contemporary society. Therefore, such processes seem to suggest the need for a more thorough analysis of the emotional foundations of economic life.

## Data availability statement

The raw data supporting the conclusions of this article will be made available by the authors, without undue reservation.

## Author contributions

The author confirms being the sole contributor of this work and has approved it for publication.

## Conflict of interest

The author declares that the research was conducted in the absence of any commercial or financial relationships that could be construed as a potential conflict of interest.

## Publisher's note

All claims expressed in this article are solely those of the authors and do not necessarily represent those of their affiliated organizations, or those of the publisher, the editors and the reviewers. Any product that may be evaluated in this article, or claim that may be made by its manufacturer, is not guaranteed or endorsed by the publisher.
